# Efficient preparation of kaolinite/methanol intercalation composite by using a Soxhlet extractor

**DOI:** 10.1038/s41598-019-44806-y

**Published:** 2019-06-06

**Authors:** Hao Qu, Sihui He, Haiquan Su

**Affiliations:** Inner Mongolia Key Laboratory of Chemistry and Physics of Rare Earth Materials, School of Chemistry and Chemical Engineering, Inner Mongolia University, Hohhot, 010021 China

**Keywords:** Materials chemistry, Organic-inorganic nanostructures

## Abstract

Kaolinite/methanol intercalation composite (KMe) is a key precursor for preparing clay-based inorganic/organic hybrid materials and kaolinite nanoscrolls. However, synthesis of KMe is a time and methanol dissipative process and the complexity of this process also limits its further applications. In this study, Soxhlet extractor was introduced to synthesize an intercalation composite and KMe was efficiently synthesized in a Soxhlet extractor through a continuous displacement process by using kaolinite/DMSO intercalation composite (KD) as a precursor. The formation process of kaolinite/methanol intercalation composite was studied by X-ray diffraction (XRD) and infrared spectroscopy (IR). The results showed that the DMSO in kaolinite could be completely displaced by methanol in this process and the preparation of KMe could be completed in 8 hours, which was far faster than the reported methods. Moreover, methanol used in this process could be recycled. Furthermore, the resulting material could be successfully used to prepare kaolinite nanoscrolls in high yield.

## Introduction

Researches on organic-inorganic hybrid materials have been thriving over the past few years because these materials combined the structural, physical and chemical properties of both inorganic host materials and organic guest species^1–3^. Intercalation reaction of natural layered minerals is a well-known method for preparing hybrid materials in a nanoscale^4–6^. Kaolinite, a 1:1 type layered aluminosilicate, has received considerable attentions due to its natural “Janus” structure^7–10^. Several guest molecules such as dimethyl sulfoxide (DMSO), N-methyl formamide (NMF), urea (U), potassium acetate (KAc) and hydrazine can directly intercalate kaolinite^11^. These guest molecules increase the basal spacing of kaolinite which allows other molecules enter the interlayer of kaolinite to form diverse functional intercalation composites. Kaolinite/methanol intercalation composite (KMe), in which methoxy groups modified the interlayer of kaolinite, is attractive because it is an indispensable intermediate of transformation from platy kaolinite into halloysite-like nanoscroll^12–20^. Furthermore, KMe is also a versatile intermediate for further intercalation reaction of kaolinite^21^. Recently, KMe was also reported as a carrier for loading and controlled-release of anticancer drugs^22^ and amitrole^23^.

Tunney and Detellier reported KMe for the first time in 1996^24^. The reaction was carried out via a solvothermal method at a temperature of between 190 °C and 270 °C using DMSO or NMF pre-intercalators. Similar method was also adopted by Xu *et al*.^18^. The intercalation of methanol into kaolinite was performed by mixing kaolinite-DMSO pre-intercalates with a solid/liquid ratio of 1 g:75 mL in autoclaves and heating the system at 100 °C for 24 hours. Subsequently, the kaolinite nanoscrolls were achieved by the solvothermal reaction of the as-prepared KMe samples in 1 M CTAC/methanol solution. Other researchers also did amount of efforts in preparing KMe at room temperature^21,23,25–29^. However, the reaction, DMSO displaced by methanol, is very slow which usually needs one week or even longer. Moreover, the solid should be re-dispersed in fresh methanol for retreatment to displace DMSO completely, which has significantly increased the dosage of methanol. Since Y. Kuroda *et al*.^17^ reported a “One-step” exfoliation of kaolinite into nanoscroll, the preparation methods and the formation mechanism of kaolinite nanoscrolls were discussed in amount of studies^12,14–16,18,30^. However, only a few of studies were focused on the application of kaolinite nanoscrolls^31,32^ due to the bottleneck of the KMe preparation. The volatility and toxicity of methanol also perplexes the prepare operation. Thus, the simplification of preparation procedure of KMe is significantly necessary.

Traditionally, the Soxhlet extractor is a laboratory tool for continuously extracting sparingly soluble substance from a solid sample^33^. The most common application of Soxhlet extractor is to extract natural product from biomass. Recently, Soxhlet solvent extraction was reported to remove the template of ordered mesoporous material^34^. In this study, Soxhlet extraction method was introduced to prepare KMe intercalation composite. The reaction time was decreased to 8 hours and the methanol could be recycled. Furthermore, the resulting material was successfully used to prepare kaolinite nanoscrolls in high yield.

## Results and Discussion

### XRD analyses

**Figure 1:**
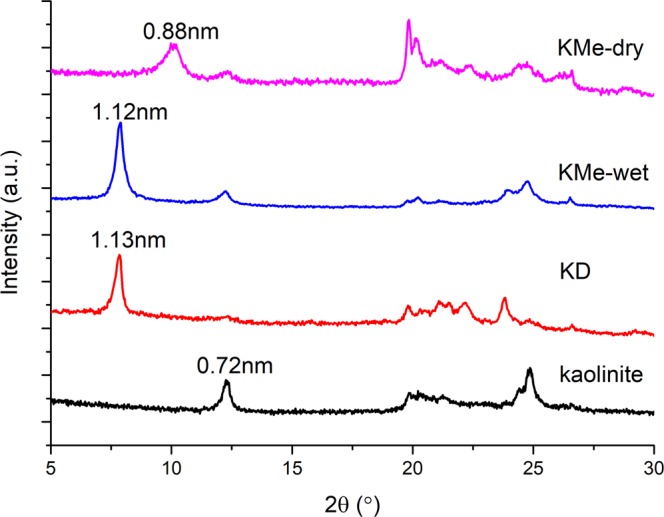
XRD patterns of kaolinite, KD and KMe.

XRD patterns of the original kaolinite and intercalated kaolinite were shown in Fig. 1 and the key data were listed in Table 1. The original kaolinite shows a basal spacing of 0.72 nm. The basal spacing expanded from 0.72 nm to 1.13 nm after intercalation of DMSO, which indicated that kaolinite/DMSO intercalated composite was successfully prepared. The intercalation ratio was calculated as high as 94.4%. A decrease of 0.01 nm of the basal spacing for KMe in a wet state was found after KD was treated for 12 hours. After drying in air for 12 hours, the basal spacing for KMe was decreased to 0.88 nm which was similar to the value (0.89 nm) reported by Zhang *et al*.^26^. The decrease of basal spacing also indicated that the intercalated methanol molecules underwent a gradual rearrangement in the interlayer of kaolinite. Deintercalation of methanol was occurred during the drying process. As a result, the intercalation ratio was reduced to 82.4%.

**Table 1:** basal spacing and intercalation ratio of different kaolinite intercalated composites.

Sample	d001/nm	Δd001/nm	Intercalation ratio/%
Kaolinite	0.72	—	—
KD	1.13	0.41	94.4
KMe-wet	1.12	0.40	85.7
KMe-dry	0.88	0.16	82.4

Intercalation ratio = I_i(001)_/(I_i(001)_ + I_k(001)_), where I_i(001)_ and I_k(001)_ are the (001) peak intensities of intercalated composites and kaolinite, respectively.

### IR analyses

**Figure 2:**
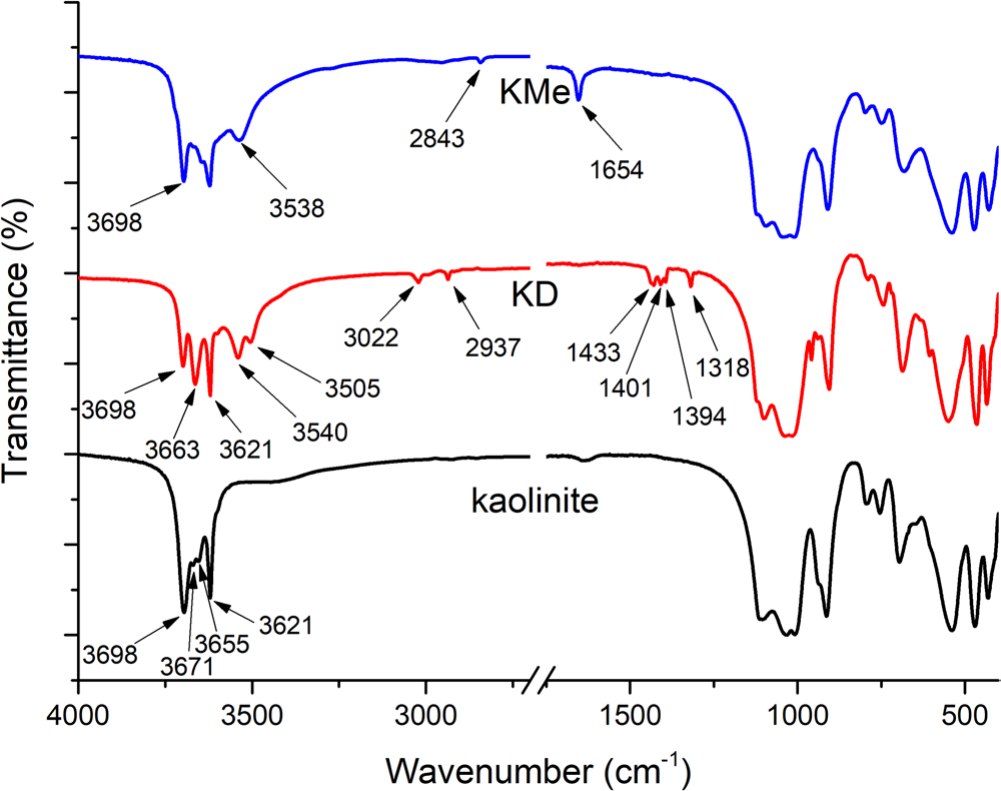
IR spectra of kaolinite, KD and KMe.

The IR spectra were normalized by the intensity of the strongest peak and shown in Fig. 2. Kaolinite shows four characteristic OH-stretching bands at 3698, 3671, 3655 and 3621 cm^−1^, and three of them at 3698, 3671, 3655 cm^−1^ were attributed to the three stretching modes of the outer Al-OH groups and 3621 cm^−1^ band was assigned to the stretching of inner Al-OH groups^35^. After the intercalation of DMSO, the typical hydroxyl bands at 3698 and 3621 cm^−1^ were remained but the intensity of 3698 cm^−1^ band decreased. Meanwhile, a series of new bands were found in the IR spectrum of KD. Bands at 3022 and 2937 cm^−1^ were the stretching vibration absorption of -CH_3_ groups and the bands at 1433, 1401, 1394 and 1318 cm^−1^ were corresponding to bending vibration in DMSO molecules. The emerging of 3540 and 3505 cm^−1^ bands and the intensity weakening of 3698 cm^−1^ band suggested the formation of hydrogen bond between outer Al-OH of kaolinite and S=O groups of DMSO^36^. As a result, two asymmetric stretching vibrations modes of Al-OH at 3671, 3655 cm^−1^ degenerated into one at 3663 cm^−1^. These results were in good agreement with previous IR studies of kaolinite/DMSO intercalation composite^37^. The major difference between kaolinite and KMe in IR spectra is the peak at 1654 cm^−1^. This new peak could be related to C-H bending vibrations of -OCH_3_ groups^21^. Accordingly, a new weak peak at 2483 cm^−1^ was detected in KMe, which indicated the presence of -CH_3_ group. Meanwhile, the peak intensity of the outer Al-OH groups at 3698 cm^−1^ decreased in the spectrum of KMe compared with that in kaolinite. These changes suggested the covalent modification of -OCH_3_ group and the formation of Al-O-C bond between outer Al-OH and methanol, which was verified by Li *et al*.^28^. In addition, a new peak observed at 3538 cm^−1^ suggested the formation of hydrogen bond between methanol and un-grafted outer Al-OH^28^, which also indicated the intercalation of methanol. The vanished peaks at 3505, 3022, 2937, 1433, 1401, 1394 and 1318 cm^−1^ belonged to DMSO demonstrated that the intercalated DMSO molecules were completely displaced by methanol molecules. In brief, XRD and IR analyses confirmed the formation of KMe.

### The effect of reaction time

**Figure 3:**
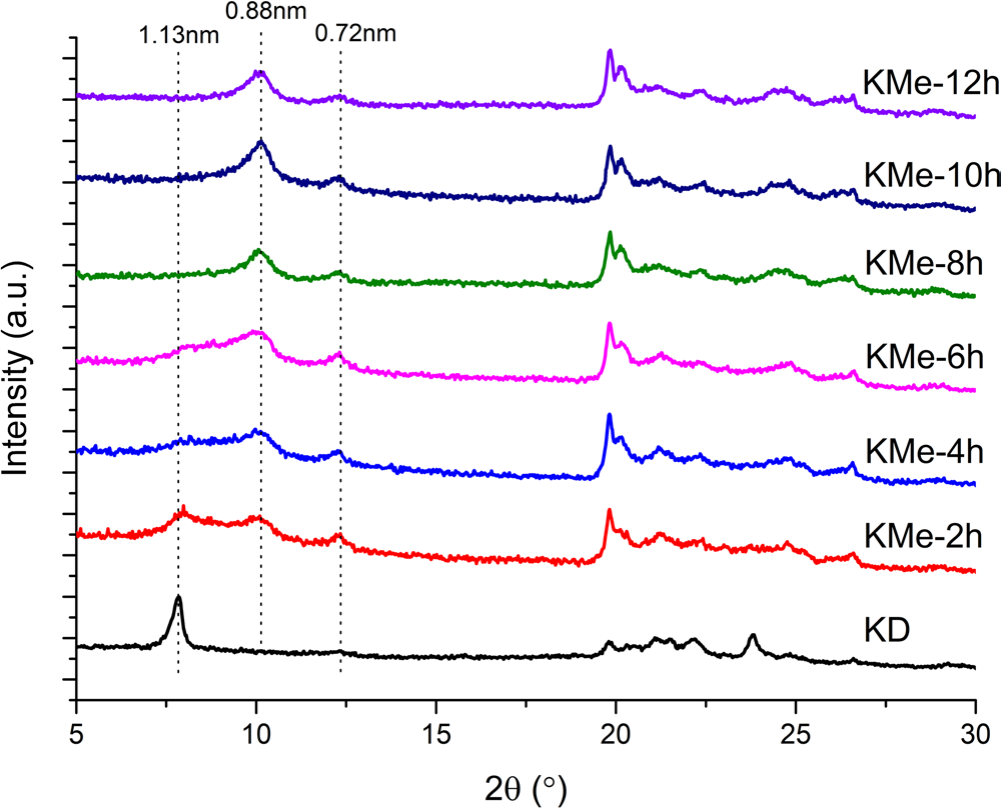
XRD patterns of KD powder treated in methanol for different time.

**Figure 4:**
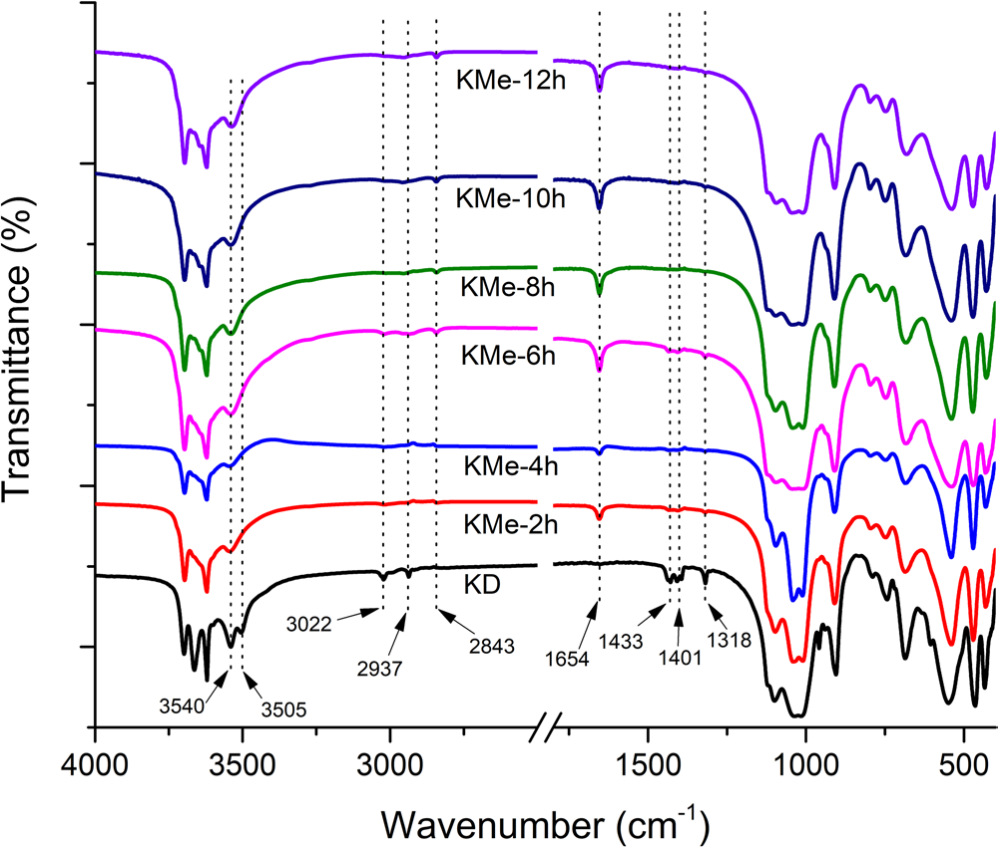
IR spectra of KD powder treated in methanol for different time.

The effect of reaction time on intercalation degree was also investigated by XRD and IR. XRD patterns of KMe samples at different reaction time were shown in Fig. 3. With the increase of reaction time, the (001) peak intensity of methanol intercalation composite (0.88 nm) became stronger but that of DMSO intercalation composite (1.13 nm) showed an opposite tendency. IR spectroscopy is a more sensitive characterization method than XRD. The dependence of IR spectra changes on the reaction time of KD powder treated in methanol was shown in Fig. 4. The increasing intensity of bands at 1654 cm^−1^ in the first 8 hours indicated the gradually formation of Al-O-C. After 8 hours of reaction, the characteristic peaks of DMSO could no longer be detected and significant changes between the spectra could not be observed. Combining the results of XRD and IR, it could be identified that DMSO molecules were completely displaced by methanol in 8 hours.

### Probable reaction process

The reaction in the extractor could be described by Eq. (1). Concretely, KD was prepared as the reaction precursor in which DMSO molecules increased the basal spacing of kaolinite. Then, displacement procedure of methanol was conducted in the chamber. Methanol steam was cooled by cooling water and the cooled liquid methanol with a temperature of 65 °C dropped into the chamber. The intercalated DMSO molecules bonded with outer Al-OH of kaolinite by hydrogen bonds were partially replaced by methanol molecules. Further, according to the IR results, one methanol molecule reacted with one outer Al-OH group, forming one Al-O-C bond and one molecule of H_2_O. The liquid mixture of methanol, H_2_O and DMSO flowed into the flask after the chamber was filled up and one displacement cycle was accomplished. The cooled fresh methanol could participate in another cycle of reaction but H_2_O and DMSO maintained in the flask owing to the boiling point gaps among methanol, H_2_O and DMSO. The reaction ended up when almost all DMSO molecules are displaced completely. The fresh methanol molecules continuously decreased the concentration of H_2_O and DMSO on the surface of kaolinite, which promoted the chemical equilibrium of Eq. (1) and increased reaction rate.

1 $${{\rm{K}}}_{{\rm{surface}}}{\rm{OH}}\ldots {{\rm{DMSO}}}_{{\rm{chamber}}}+{{\rm{MeOH}}}_{{\rm{fresh}}}\to {{\rm{K}}}_{{\rm{surface}}}{{\rm{OMe}}}_{{\rm{chamber}}}+{{\rm{H}}}_{2}{{\rm{O}}}_{{\rm{flask}}}+{{\rm{DMSO}}}_{{\rm{flask}}}$$

### Comparison with other preparation methods

Preparation methods of KMe adopted by reported literatures were listed in Table 2. It could be seen from the table that the high temperature solvothermal method could accelerate the reaction and shorten the reaction time. But it still takes one day or longer to complete this reaction. In the case of stirring method at ambient temperature, the reaction time takes longer than one week. Since the replacement reaction once reaches equilibrium, the reaction is difficult to proceed. Therefore, the preparation of KMe by the conventional method requires repeated replacement of fresh methanol, which leads to a large increase dosage of methanol in the preparation process and the spent methanol is difficult to be recycled. Compared with the reported methods^18,21,22,24,26,29^ listed in Table 2, our method reduced preparation time significantly. Moreover, the spent methanol could be reused in another preparation cycle, which decreased methanol dosage as well.

**Table 2:** Preparation conditions of kaolinite/methanol intercalation composite.

Method	Precursor	Precursor mass/g	Temp./°C	Time/h	Total methanol dosage/mL	Ref.
S.E.	KD	5	65	8	100	This work
S.T.	KD	10	200–230	35	30	^21^
S.T.	KD	1	100	24	300	^15^
S.R.	KNMF	—	a. t.	168	—	^26^
S.R.	KD	5	a. t.	240	1200	^23^
S.R.	KD	20	a. t.	192	4040	^18^
S.R.	KD	5	a. t.	168	100	^19^

S.E. = Soxhlet extraction; S.T. = Solvothermal; S.R. = Stirring; a.t. = ambient temperature.

### Preparation of kaolinite nanoscrolls

**Figure 5:**
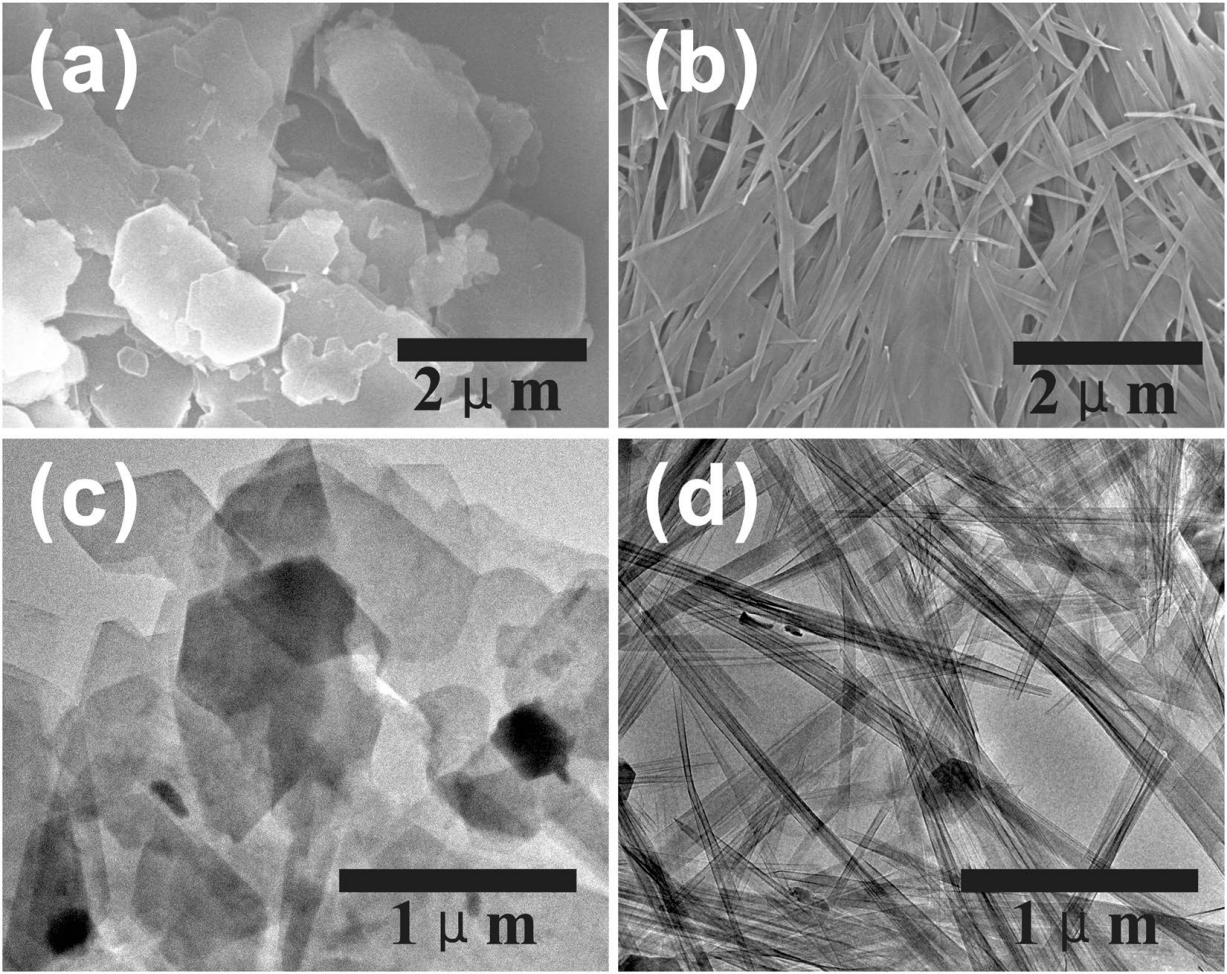
SEM images of (**a**) kaolinite, (**b**) kaolinite nanoscrolls and TEM images of (**c**) kaolinite, (**d**) kaolinite nanoscrolls.

**Figure 6:**
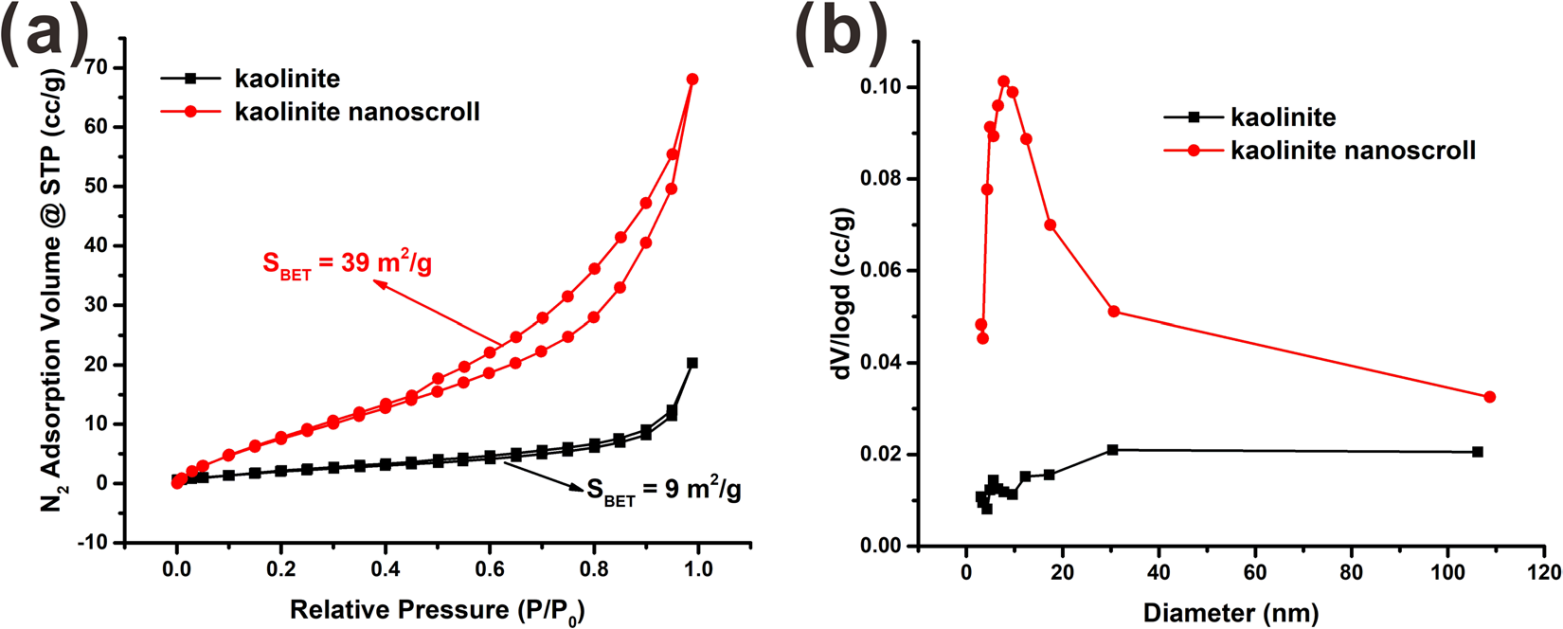
(**a**) N_2_ adsorption-desorption curves and (**b**) pore size distribution curves of kaolinite and kaolinite nanoscrolls.

The KMe which was obtained by treating the KD with methanol in the extractor for 12 hours (KMe-12h) was applied in preparing kaolinite nanoscrolls. The morphology of kaolinite and kaolinite nanoscrolls was studied by SEM and TEM. The original kaolinite presented a pseudo-hexagonal plate-like morphology with a size of 0.2 to 2 μm (Fig. 5a,c) which indicated the well-crystallinity of the original kaolinite. However, most of the kaolinite sheets were transformed from plates into halloysite-like nanoscrolls after solvothermal treatment (Fig. 5b,d). The length of kaolinite nanoscrolls was around 2 μm which depended on the diameter of the original kaolinite sheet and the inner diameter of the nanoscrolls was around 20 nm. It is significantly important that only very few uncurled kaolinite sheets were found because of the efficient intercalation of methanol. Thus, the yield of kaolinite nanoscrolls prepared by this method is much higher than that reported in the literature^14,17,18^. The transformation mechanism was discussed by Li *et al*.^16^ and could be summarized as two aspects: On the one hand, the size discrepancy between Si-O tetrahedron and Al-O octahedron results in the crimping of kaolinite sheets into nanoscrolls. On the other hand, the displacement intercalation of CTAC molecules exfoliated layers of kaolinite and promoted the platy kaolinite sheets to be curved into nanoscrolls spontaneously. Great changes on the textural properties of kaolinite were found after the platy sheets crimped into nanoscrolls. As shown in Fig. 6a, the original kaolinite exhibited a type II isotherm with an H_3_ hysteresis loop according to the classification of IUPAC, which indicated that kaolinite was a non-porous solid. The specific surface area of kaolinite was calculated as low as 9 m^2^/g. On the contrary, the type IV isotherm and H_2_ hysteresis loop of kaolinite nanoscrolls suggested a mesoporous structure of the sample. Meanwhile, the specific area of kaolinite nanoscrolls was increased to 39 m^2^/g, which was more than 4 times larger than that of the original kaolinite. Moreover, the original kaolinite showed a wide pore size distribution from micropore to macropore while the kaolinite nanoscrolls revealed a narrow pore size distribution of 5 to 30 nm (Fig. 6b), which was in accordance with the TEM analysis. The uniform pore structure of kaolinite nanoscrolls would make the material have potential applications in the fields of catalysis, adsorption, etc.^38–43^.

## Methods

### Materials

Kaolinite (natural) was purchased from Sigma-Aldrich. Dimethyl sulfoxide (DMSO, AR) and anhydrous methanol (MeOH, AR) were purchased from Tianjin Fengchuan Chemical Reagent Factory and used as obtained. Hexadecyltrimethylammonium Chloride (CTAC, CP) was purchased from Sinopharm Chemical Reagent Co., Ltd. All of the reagents were used without further purification.

### Sample preparation

Kaolinite/DMSO intercalated composite was prepared according to literature. 10.0 g Kaolinite powders were dispersed in the mixture of 90 mL DMSO and 10 mL H_2_O. Then, the suspension was magnetically stirred at 80 °C for 24 hours. Solid particles were separated by centrifugation, washed with anhydrous ethanol twice and dried at 60 °C for 12 hours. The resulted powder was labeled as KD.

**Figure 7:**
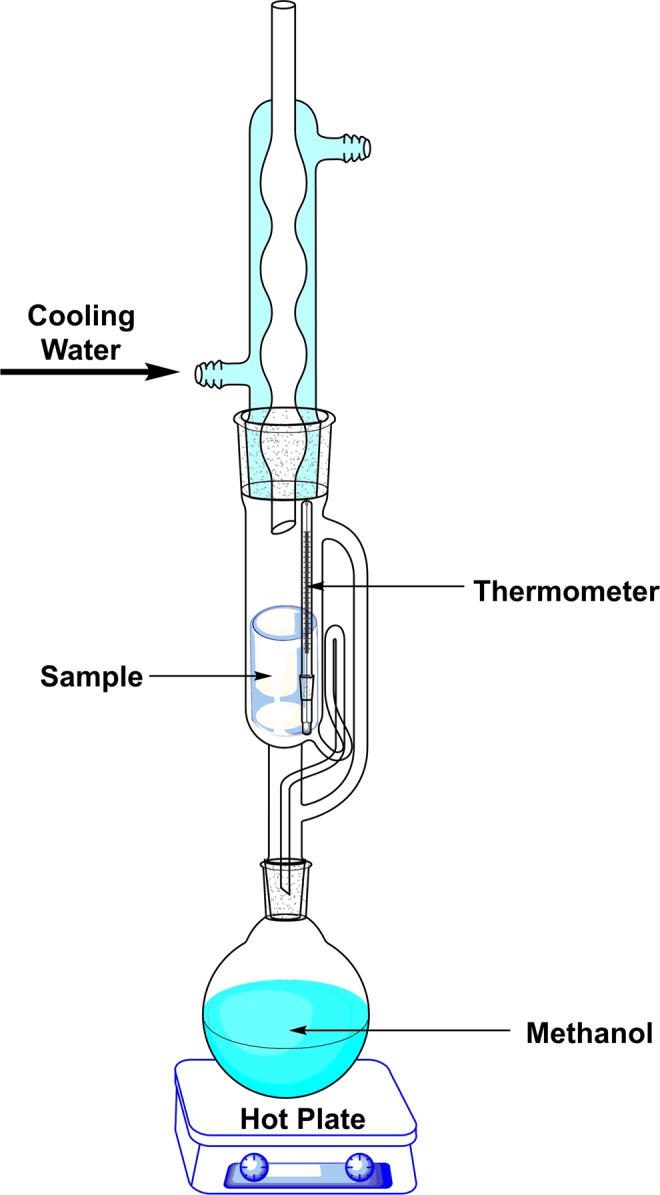
Displacement reaction in a Soxhlet extractor.

The kaolinite/methanol intercalated composite was synthesized through a continuous displacement process in a Soxhlet extractor. The experimental setup was shown in Fig. 7. 5.0 g KD was wrapped in a filter paper and putted into the Soxhlet chamber. A thermometer was putted in the chamber to measure the reaction temperature. 100 mL Methanol was poured into the flask and heated to boil. Cooling water was flowed before heating. Methanol in the chamber was cycled about every 15 min and the reaction temperature was 65 °C, which was similar to the boiling point of methanol. The reaction was performed for 12 hours and sample was collected every 2 hours for XRD and IR analysis during the reaction. The final product was dried in air for 12 hours and named as KMe.

Kaolinite nanoscrolls was prepared according to the literature^18^ with a little modification. 1.0 g wet KMe was dispersed in 75 mL 1 M CTAC/methanol solution. The dispersion was transferred into a 100 mL PTFE-lined autoclave, which was sealed and heated at 120 °C in an oven for 24 hours. The ultimate product was washed three times by ethanol and collected by centrifugation.

### Characterization methods

X-ray diffraction (XRD) patterns were obtained from a PANalytical Empyrean X-ray diffractometer using CuKα radiation (λ = 1.54059 Å) at 40 kV and 40 mA. Fourier transform infrared spectroscopy (FT-IR) analysis was carried out with a Thermo Scientific Nicolet 6700 FT-IR spectrometer to determine the functional groups in all of the samples. Samples were ground with KBr into fine powders and the homogeneous mixture was pressed into a disk. FT-IR spectra within the wavenumber range of 400–4000 cm^−1^ were obtained by averaging 32 scans at a resolution of 2 cm^−1^. The morphological images of samples were recorded using a Hitachi S-4800 Scanning electron microscopy (SEM) and a FEI Tecnai G2 F20 field-emission transmission electron microscope (TEM). The textural properties of samples were characterized by the N_2_ adsorption-desorption test performed on a Quantachrome Autosorb iQ2 MP analyzer. The specific surface area and pore size distribution of samples were calculated by Brunauer-Emmett-Teller (BET) and Barrett-Joyner-Halenda (BJH) method, respectively.
